# Finite element analysis and in vitro simulation experiments on ophthalmic trocar needles

**DOI:** 10.3389/fbioe.2025.1738029

**Published:** 2026-01-12

**Authors:** Jiexin Sun, Zezhong Zhang, Hailun Yuan, Gaiping Zhao

**Affiliations:** 1 School of Health Science and Engineering, University of Shanghai for Science and Technology, Shanghai, China; 2 Sierra Medical System (Suzhou) Co., Ltd., Suzhou, China

**Keywords:** biomechanics, finite element analysis, *in vitro* simulation experiment, ophthalmic trocar systems, trocar needles

## Abstract

**Introduction:**

Ophthalmic trocar systems are commonly used to establish a passage into the vitreous cavity in complex ophthalmic surgeries, and optimizing the design of trocar needles may potentially reduce surgical trauma and the risk of complications.

**Methods:**

Study combined *in vitro* simulation tests with finite element analysis, in details, four trocar needles with varying outer diameters (23G and 25G), bevel lengths and tip quadrilateral surface areas were evaluated. *In vitro* penetration force tests and penetration force measurement were performed to explore the impact of each trocar needles’ parameters on its performance, while finite element analysis was introduced to reveal phasic characteristics of stress distribution in scleral tissue during needle penetration and correlation between stress distribution and the needle tip structure.

**Results:**

In penetration force tests, the penetration force of the 25G needles was 14.62% lower than that of the 23G group (P < 0.001), cause a smaller needle diameter increased penetration efficiency. Further findings showed that the 23G needles with a smaller needle tip surface and longer bevel had a significantly lower penetration force than the 25G needle (P < 0.01), which indicated that bevel length along with the tip surface play vital roles in penetration efficiency. The penetration force measurement results showed that an optimal range of tip quadrilateral surface areas led to better tip strength performance. Finite element analysis found that the initial stress concentration was primarily determined by the geometry of the needle tip, while the diameter and surface properties of the needle shaft influence the stress distribution throughout the penetrating process, which interpreted the data *in vitro*. The smaller tip area led to more concentrated stress, and a longer bevel can distribute stress, reduce resistance during the penetration process and improve penetration efficiency.

**Discussion:**

This study proved that a multi-stage tapering needle with apex truncated and a reasonable bevel length enhanced the strength of the needle tip while improving cutting efficiency, and provided scientific basis for designing ophthalmic trocar systems. Clinical studies could be taken in the further to meet the ophthalmic surgery developing toward greater precision and minimal invasiveness.

## 1 Introduction

Ophthalmic surgeries aim to repair, reconstruct, or replace ocular tissues affected by disease or injury. Procedures such as retinal surgery, vitrectomy, and cataract surgery target intraocular tissues including the lens, retina, and vitreous. These operations represent highly precise intraocular interventions, requiring access to the ophthalmic cavity through small incisions for diagnostic or therapeutic procedures (Williamson, 2021; He et al., 2021). In complex ophthalmic surgeries, ophthalmic trocar systems are commonly used to establish a passage into the vitreous cavity, avoiding the damage to the ocular tissues caused by the repeated entry and exit of surgical instruments. The design of the ophthalmic trocar system is closely related to its effectiveness and safety in clinical apply (Sabti and Raizada, 2022; Davidović et al., 2024).

Vitrectomy is an ophthalmic surgical procedure for treating a range of vitreous and retinal diseases (Gupta et al., 2018). Its primary purpose is to remove opacified vitreous or vitreoretinal traction, thereby restoring transparent refractive media, facilitating retinal reattachment, and ultimately improving the patient’s visual function. Access to the vitreous cavity is achieved through conjunctival or scleral incisions by using special medical instruments to remove the abnormal vitreous tissue (Williamson, 2021). Trocar systems are employed to establish access ports into the vitreous cavity in order to avoid repeated entry and exit of surgical instruments, which causes ocular trauma. Typically, three ports are created: one for infusion, one for vitrectomy, and one for other procedures such as laser therapy or retinal reattachment (Lim and El-Amir, 2016). Given the small size of the human eye (24–25 mm in diameter) and its complicated network of nerves and blood vessels, ophthalmic surgical instruments are typically small in size (Mercanti and Renna, 2017). Therefore, ophthalmic trocars are far smaller than laparoscopic counterparts (3–15 mm outer diameter, OD). Their diameter has gradually reduced from the initial 17G to 20G and 23G (OD ≈ 0.7 mm, the current mainstream size). This reduction has markedly decreased tissue trauma and surgical complications (Kim et al., 2007), and usually the wound can close on its own without sutures.

Given the unique anatomical characteristics of eyes, ophthalmic trocar systems require more stringent design and manufacturing standards. Over multiple iterations, trocars for minimally invasive vitreoretinal surgery have been refined in terms of tip geometry, diameter size, the emergence of valves, penetration force, cutting efficiency, wound shape after penetration, and the ease of surgical handling (Marques et al., 2015). At present, more than 10 types of ophthalmic trocar systems are available on the market, including mainstream ones such as Alcon Entry System Enhanced, Bausch and Lomb Loaded Clampshell PMP part: S-APX-B + L ESA, DORC One Step Cannula System, etc. (Meyer et al., 2014), so that only a limited number of products are commercially available and there is still a need for systematic strategies to optimize the structure of each part of the trocar systems.

Finite element analysis (FEA) breaks down complex structures into a finite number of simple elements, for effectively simulating, calculating, analyzing, and predicting the mechanical behavior of tissues, including stress distribution, deformation, and the interaction between instruments and tissues. It has been widely used in the simulation of surgical punctures (DiMaio and Salcudean, 2003). Computer modeling of ophthalmic surgery has also shown great potential in ophthalmology, which can simulate surgical procedures and predict complications. Also, as an important tool for the development and optimization of ophthalmic instruments it not only improves design accuracy and efficiency but also significantly reduces R&D costs and ethical risks (Schutte et al., 2006; Pang et al., 2024; Foster et al., 2022). Previous research has shown that different parameters of trocar needles’ geometry collectively affect penetration efficiency, incision morphology, and tissue damage severity in clinical settings, and significant differences in puncture force and cutting effect among 23G trocar systems with different needles’ geometry were observed (Fujii et al., 2002). Thus, a better geometric design of trocar needles can enhance surgical safety and effectiveness (Kim et al., 2007). This study combines FEA with *in vitro* simulation tests to optimize the design of the ophthalmic trocar systems, better reducing surgical trauma and the risk of complications.

## 2 Materials and methods

### 2.1 Ophthalmic trocar systems

This study researched four commercially available ophthalmic trocar systems and four Sierra-developed ones (Table 1). Prior to *in vitro* penetration force tests and tip strength tests, all needle tips in their original state underwent meticulous inspection to detect any unintended manual damage under a microscope after opening the package and removing protective caps.

**TABLE 1:** Geometric parameters, materials, and products schematic representation of eight ophthalmic trocar systems and their needles.

Manufacture	Description	Size, Gauge	Bevel type	Primary angle (a), in degrees	Point length, mm	Bevel length, mm	Material of cath	Magnified schematical drawing
Alcon	Entry system enhanced	23G	Spear	12.7 (12.6–12.8)	2.71 (2.60–2.82)	1.55 (1.49–1.62)	Metal	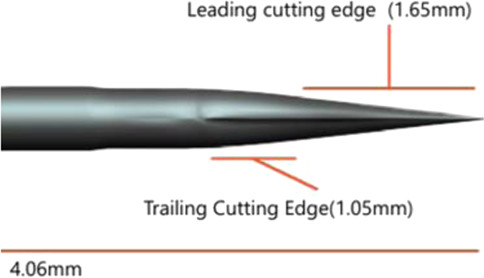
Alcon	Entry system enhanced	23G	Spear	11.2 (11.0–11.4)	2.20 (2.18–2.22)	1.07 (1.05–1.08)	Metal	
DORC	One step cannula system	23G	Spear	13.3 (13.1–13.5)	3.11 (3.05–3.16)	-	Metal	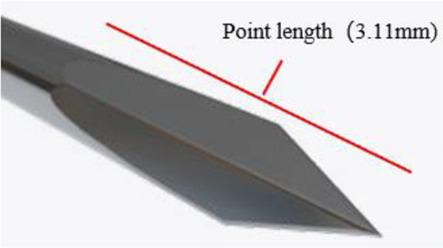
DORC	One step cannula system	25G	Spear	12.3 (12.0–12.5)	2.79 (2.74–2.84)	-	Metal	
Sierra	Disposable trocar systems 23G (Group A)	23G	Spear	13.2 (12.9–13.5)	3.17 (3.13–3.22)	1.11 (1.08–1.12)	Stainless steel 302	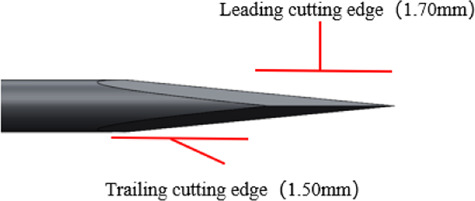
Sierra	Disposable trocar systems 23G (Group B)	23G	Spear	13.3 (13.1–13.5)	2.88 (2.78–2.98)	1.47 (1.45–1.5)	Stainless steel 302	
Sierra	Disposable trocar systems 25G (Group A)	25G	Spear	12.4 (12.2–12.6)	2.86 (2.81–2.91)	1.02 (0.96–1.09)	Stainless steel 302	
Sierra	Disposable trocar systems 25G (Group B)	25G	Spear	12.4 (12.2–12.6)	2.35 (2.33–2.37)	1.27 (1.12–1.43)	Stainless steel 302	

The tip designs of trocar needles have various types, including back bevel, spear bevel, lancet bevel, and spatula bevel (La Chapelle, 2015). Alcon Entry System Enhanced is spear bevel, while Bausch and Lomb feature spatula bevel. A study has shown that back bevel, spear bevel, and lancet bevel have lower manual penetration force than the spatula bevel design. However, the linear incisions formed by the lancet bevel and spear bevel trocar needles facilitate wound closure (Meyer et al., 2014). In comparison, incisions formed by the spatula bevel needle may increase the risk of postoperative leakage due to unstable wound structure. Entry System Enhanced trocar systems manufactured by Alcon, featuring spear bevel needles and linear incision, hold a significant market share due to their high penetration efficiency and enhanced safety.

**FIGURE 1:**
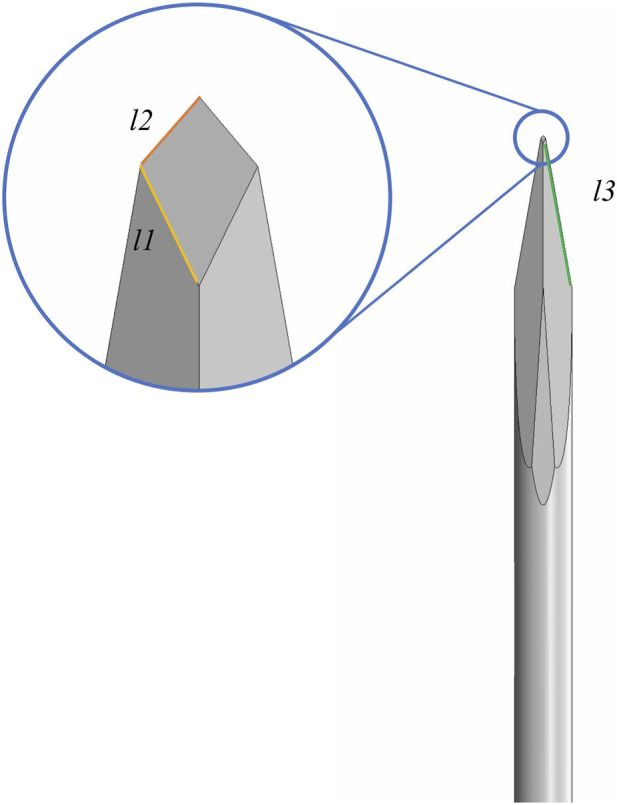
Magnified schematical drawing of Sierra 23G,25G needle.

In contrast to commercially available products, four Sierra disposable trocar systems have an innovative design that integrates spear bevel tips with a multi-stage tapering structure. This is because the spear bevel tip features sharpness and uniform force distribution. By combining with a multi-stage tapering design, this innovative tip design can precisely disperse penetration force upon contact with scleral tissue, avoiding excessive tissue damage caused by stress concentration (Jiang et al., 2016). Additionally, an apex-truncated tip structure is introduced to further enhance needle tip strength, and the range of apex-truncated tip structure parameters of Sierra products was obtained through extensive quantitative testing and comparative analysis, ensuring that the spear bevel tip maintains its sharpness and consistent penetration performance over repeated use. The four Sierra trocar needles have different bevel lengths Figure 1. Illustrates the structural schematic of a trocar needle tip, where *l3* represents the bevel length and *l1* and *l2* represent the two edges of its quadrilateral facet. The *l1*, *l2* and *l3* values of each trocar needle were measured under ×100 magnification. The 4 needle tips are categorized into two groups according to the variations in edge lengths: Group A, where *l1* and *l2* range from 0.14 ± 0.02 mm and 0.17 ± 0.02 mm, respectively; and Group B, where *l1* and *l2* range from 0.04 ± 0.02 mm and 0.06 ± 0.02 mm, respectively. All four Sierra disposable trocars share the same external design but have two different outer diameter sizes, 23G and 25G. So based on the two OD sizes, Groups A and B are further divided into four subgroups: 23G (Group A), 23G (Group B), 25G (Group A), and 25G (Group B).

### 2.2 *In Vitro* penetration force tests

#### 2.2.1 Penetration force measurement method

**FIGURE 2:**
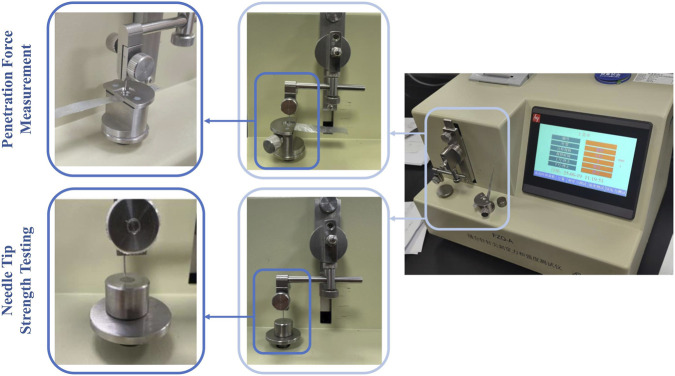
Trocar needles performance testing method.

This study conducted penetration force tests on the needle tips in accordance with the DIN 13097 standard. Aluminum foil with a thickness of 0.05 ± 0.002 mm, purity ≥99.5%, tensile strength ≥3 kg/mm^2^, and elongation ≥3% was selected as the test medium, an industry-recognized material used for objectively evaluating manufacturing consistency and geometric sharpness of needle tips. It was fixed by using an aluminum foil fixture and ensured no initial tensile or compressive stress. Needle samples from the four Sierra disposable trocar groups (23G-A, 23G-B, 25G-A, 25G-B; number = 30 per group) were vertically fixed by a needle holder and driven toward the foil at a constant velocity ≤10 mm/s. The peak penetration force was automatically recorded at the moment the needles pierced the foil and triggered the electrode (Figure 2). After each test, the instrument was reset and the aluminum foil was moved to ensure that the spacing between adjacent penetration places was greater than three times the needle’s diameter. Each sample was tested three times and the mean value was taken as the penetration force indicator for that sample. The parameters of the test instruments met the following specifications: full-scale range 1.2 N, minimum readable value 0.01 N, which meet standardized accuracy requirements.

#### 2.2.2 Needle tip strength testing method

Needle tip strength is defined as the critical load at which tip bending deformation occurs. During testing, the trocar needle was clamped by a needle holder and approached a steel block surface vertically at a speed of ≤10 mm/s. According to ISO standards for load requirements of 23G and 25G trocar needles, when the chord length L ≥ 12 mm, the target load is set to 0.58 N and the load should be maintained for 5–10 s before it is released. To observe the origine (pre-test)morphological differences between four trocar needles precisely, a ×170 magnification (SangNond Micro Measurement Electron Microscope SN-6000M) was selected. The post-test trocar needle was observed using a ×5 magnifying glass as specified in the ISO standard, or by dragging the needle tip across cotton wool. The absence of fiber pull-out indicates no tip bending deformation.

### 2.3 Finite element analysis

#### 2.3.1 Model establishment

**FIGURE 3:**
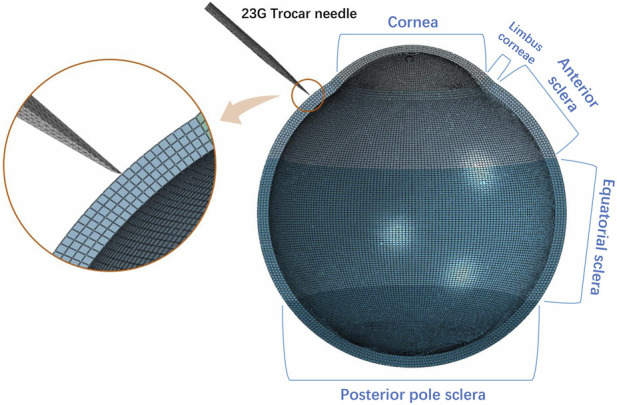
23G Trocar needle - Sclera Coupled Model.

In this study, a trocar-sclera coupled model was developed by using finite element to address nonlinear material response and localized large deformation during insertion (Figure 3). In this model, the geometric parameters of the 23G trocar needle (a spear-bevel tip with a quadrangular facet) were provided by Shanghai Sierra Medical Technology Co., Ltd. Corneoscleral tissue was divided into five regions: cornea, limbus, anterior sclera, equatorial sclera, and posterior sclera. The pars plana, which represents parts of the anterior sclera and is the primary clinical insertion site, was selected as the target analysis region.

Given the fact that stress concentration is around the needle tip, a symmetrically structured mesh refinement strategy was employed to divide the region from the needle tip to the entire scleral layer (approximately 1.2 mm thick) into hexahedral meshes, with element sizes controlled between 60 and 80 μm. This refinement level is justified to ensure sufficient resolution for accurately capturing key mechanical responses, such as peak stress and penetration initiation behavior, within the focal region of needle-tissue interaction. While the peripheral tissue region was modeled with unstructured meshes as a transition, with a total number of approximately 80,000. This mesh design effectively enhances computational solving efficiency. The penetration process was simulated by using a dynamic explicit solver (loading speed of 10 mm/s, consistent with the *in vitro* experiments). It focused on capturing the penetration force-displacement curve and the characteristics of principal stress distribution.

After analyzing the mesh size sensitivity, the local mesh size for the tip and tissue contact region was determined to be 0.08 mm, which ensured both computational efficiency and an accurate representation of crack evolution. Furthermore, to simulate the local failure of the scleral tissue during the needle penetration process, this study developed a VUSDFLD subroutine based on the Von Mises stress failure criterion to control the deletion of failed elements. The scleral tissue region and the needle were represented by C3D8RH and rigid elements, respectively. Their material properties are detailed in Table 2.

**TABLE 2:** Material properties of the finite element model.

Parameter	Sclera	Trocar needle
Material model	First order ogden	Stainless steel 302
Material parameters	$\mu_{1}=0.271,\alpha_{1}=150.0$	E=193GPa,v=0.25
Density	0.96 g/cm^3^	7.86 g/cm^3^

#### 2.3.2 Boundary condition setting

The process of the needle penetrating the scleral tissue involves multiple contact forms, including interactions between the needle and the tissue’s outer surface, tissue’s interior, its own surface, and interaction between different elements of the sclera. The contact between the needle tip and the tissue outer surface was modeled as surface-to-surface contacting, with a hard contact in the normal direction and a Coulomb friction model combined with a finite slip algorithm in the tangential direction. Since internal tissue damage may lead to element-to-element contact, it was necessary to define internal contacts. To this end, element sets and reference surfaces were first created in the Abaqus pre-processing module to specify contact regions. After exporting the input file, key commands were manually inserted to specify the internal contact surfaces. Six contact pairs were defined, with hard contact in the normal direction and a friction coefficient of 0.4 (Zahedi et al., 2014), in order to simulate the actual frictional behavior during puncture. In addition, fixed boundary conditions were applied to both internal and external surfaces of the scleral tissue model to restrict all degrees of freedom. As to the issues of a large number of models meshes and high computational costs, a mass scaling factor of 1,000 was applied to the entire model before the analysis step, which increases the stable time increment, improve computational efficiency, and ensure stable calculation results.

## 3 Results

### 3.1 Penetration force tests

A total of 120 needles from 4 groups of disposable trocars, including 23G (Group A), 23G (Group B), 25G (Group A), and 25G (Group B), were subjected to penetration force tests and all groups demonstrated good measurement repeatability. The overall mean penetration force for the two groups of 23G needles (Group A and Group B, n = 60) was 0.472 ± 0.062 N, while the overall mean penetration force for the two groups of 25G needles (Group A and Group B, n = 60) was 0.403 ± 0.052 N, suggesting that the penetration force of the 25G group was 14.62% lower than that of the 23G group, which indicates that the 25G needles exhibited significantly better penetration performance in the aluminum foil medium. The results of Welch’s t-test showed that the difference between the two groups means was statistically significant (t = 6.620, df = 118, P < 0.001). The 25G needles, with smaller diameter, demonstrated better penetration force than the 23G needles, which aligned with mechanical principles.

**FIGURE 4:**
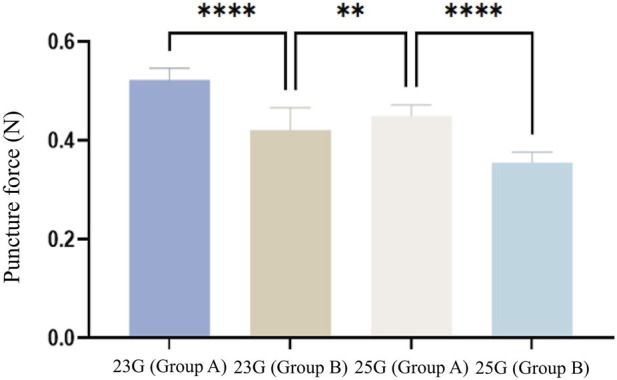
Penetration Force Test Results of Four Groups of Ophthalmic Trocar Needles (n = 120). The overall mean penetration force for 23G (Group A), 23G (Group B), 25G (Group A), and 25G (Group B) was 0.523 ± 0.023, 0.422 ± 0.045, 0.450 ± 0.023, 0.356 ± 0.021 N, respectively.

The result of the one-way analysis of variance (ANOVA) among the four groups (Figure 4) showed that between 23G (Group A) and 23G (Group B), as well as between 25G (Group A) and 25G (Group B), the penetration performance of Group B was significantly better and the differences in mean values were of great statistical significance (P < 0.001). Given the same outer diameter of the needles, the design of Group B was distinctive from Group A, with a significantly longer bevel length (P < 0.01) and smaller quadrilateral edge lengths of the needle tip (*l1* and *l2*). The differences in design may be the key factors influencing penetration efficiency. Moreover, the study found that the penetration force of 23G (Group B) was significantly lower than that of 25G (Group A) (P < 0.01). Even though a smaller needle diameter increased penetration efficiency, a comparison between parameters of 23G (Group B) and 25G (Group A) revealed that the bevel length of the 23G (Group B) needle was significantly larger than that of the 25G (Group A) (P < 0.0001), and the quadrilateral surface area of the 23G (Group B) needle was significantly smaller than that of the 25G (Group A) (P < 0.01). These findings indicate that more reasonable cutting-edge length and quadrilateral edge length of the needle tip play vital roles in penetration efficiency.

### 3.2 Needle tip strength test

**FIGURE 5:**
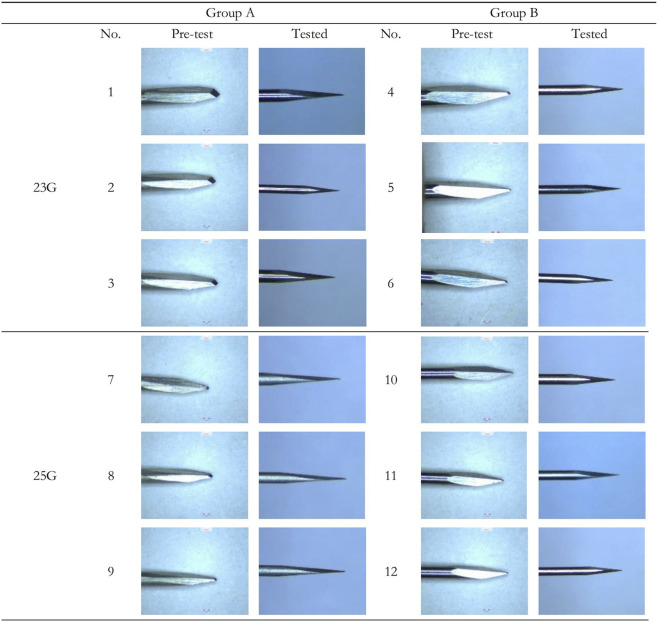
Comparison of selected trocar needles before (×170 magnification) and after testing (×5 magnification).

The needle tip strength test was to evaluate the performance of different trocar needles since variations in tip strength hugely impact their clinical performance. The test results (Figure 5 shows partial samples under ×5 magnification) indicated that the needles in Group A (23G and 25G) exhibited varying degrees of tip bending deformation and some needle tips dragged out cotton fiber during the cotton-dragging test. In contrast, the needle tips in Group B (23G and 25G) maintained intact after testing. No significant bent tip deformations were observed under ×5 magnification, and no cotton fibers were pulled out. Thus, Group B needles with better needle tip strength performance demonstrate superior resistance to tip bending deformation compared with Group A, better meeting the strength requirements for trocar needles in clinical and practical use. Besides, parameters of the apex-truncated design differ between Groups A and B with the cross-sectional area of Group A larger than that in Group B (p < 0.0001), resulting in sharpness loss of Group A during repeated punctures. This could pose intraoperative and postoperative risks in clinical practices. Therefore, the test results suggest that optimal parameters for *l1* and *l2* of the apex-truncated needle are 0.04 ± 0.02 and 0.06 ± 0.02, respectively.

### 3.3 Finite element analysis

**FIGURE 6:**
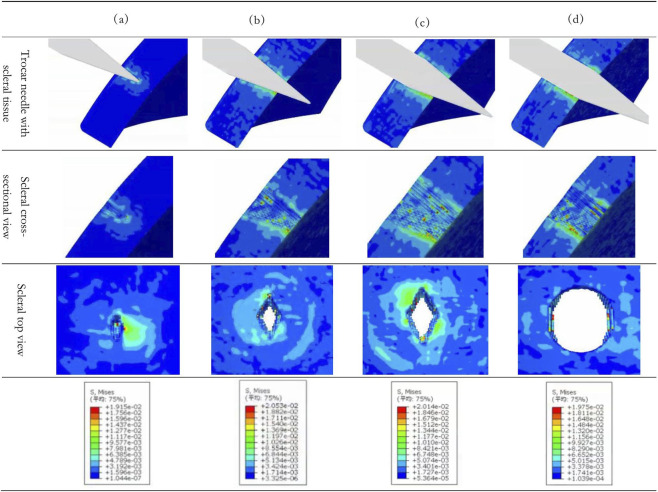
Stress Contour Maps of Scleral Tissue during Penetration with a 23G Trocar needle. **(a)** Needle tip puncturing. **(b)** Needle tip fully inserted. **(c)** Needle entering tissue. **(d)** Needle fully inserted.

To investigate the damage mechanisms of scleral soft tissue under different insertion conditions, this study presents a quantitative analysis of scleral mechanical responses based on stress contour maps obtained under four penetration scenarios (Figure 6). At the initial stage of puncture, the needle tip forms a point contact with the scleral surface. According to Hertz contact theory, the contact stress distributed along the needle shaft in a gradient pattern, forming a pyramidal region of stress concentration. At this point, the peak equivalent stress reached 19.15 kPa. Due to the geometric amplification effect caused by pyramidal structure of the needle tip, localized stress density at the edges increased significantly, resulting in a wound shape that corresponded closely to the cross-sectional geometry of the needle tip. This stress concentration phenomenon not only determined the initial location of the injury but also laid the foundation for stress transmission during subsequent penetrations.

As the needle slowly penetrates the sclera, the stress distribution maintained its conical characteristics, but when the peak stress rose to 20.14 kPa, the stress concentration zone gradually shifted to the inner surface of the sclera. This phenomenon was caused by the mechanical effects during needle advancement: the tissue in front underwent plastic deformation due to compression, while the tissue behind experienced stress relaxation delay due to viscoelasticity, leading to continuous stress accumulation on the inner scleral surface. It is worth mentioning that the shift of the stress concentration zone may introduce a risk of interlaminar tearing within the scleral layers. Based on this mechanism, a faster needle advance would allow less time for stress diffusion, theoretically intensifying inner surface damage and increasing the risk of interlaminar tearing—a premise that warrants future validation through multi-velocity studies.

After the needle completely perforated the sclera, the peak stress dropped to 19.75 kPa and the wound exhibited an elliptical entrance with a cylindrical internal channel. The elliptical entrance resulted from needle wobbling during insertion, with its long axis aligning with the tip’s wobbling trajectory. Meanwhile, the internal cylindrical channel aligned with the diameter of the needle shaft. This type of wound is prone to form stress concentrations at the edges, which can be addressed by optimizing the needle tip’s design such as adopting rounded edge as transition or reducing the diameter difference between the needle tip and the shaft in order to decrease stress concentration factors and provide a theoretical basis for minimizing postoperative complications. From a clinical perspective, this study on stress distribution disparities under varying operating conditions provides a scientific basis for selecting trocar needles and establishing operational protocols in ophthalmic surgery, thereby reducing wound stress concentration and accelerating wound healing.

By means of FEA, this study reveals phasic characteristics of stress distribution in scleral tissue during needle penetration and correlation between stress distribution and the needle tip structure. Results indicate that the initial stress concentration is primarily determined by the geometry of the needle tip, while the diameter and surface properties of the needle shaft influence the stress distribution throughout the penetrating process.

## 4 Discussion

This study focuses on optimizing the design of ophthalmic trocar needles. By combining FEA with *in vitro* simulation experiment, it explored the impact of different trocar needles’ parameters on its performance and analyzed the scientific soundness and effectiveness of their design so as to provide scientific basis for designing ophthalmic trocar systems.

To that end, the study examined four self-developed Sierra ophthalmic trocar systems of two different outer diameters (23G and 25G), which were categorized into Group A and Group B with each group featuring different lengths of quadrangular pyramid edges at the needle tip, as well as varying bevel length. According to mechanical principles, 25G trocar needles typically exhibit lower penetration forces than their 23G counterparts of identical designs. This is because 23G needles have a larger tissue contact area, which requires overcoming greater resistance during penetration, so that 23G trocar needles require higher penetration force (Tanabe et al., 2020). However, this study, obtained further insights. Results of penetration force measurements revealed that the 23G Group B needle, which has a smaller tip cross-sectional area and a longer bevel length, exhibited significantly lower penetration force than the 25G Group A needle (P < 0.01). This result of the FEA further supported the findings: during the initial stage of penetration, stress concentration was primarily determined by the tip structure. Specifically, the sharper the needle tip, the more concentrated the stress. During the middle stage of penetration, as the needle shaft gradually entered the sclera, the stress on the scleral tissue originated from the bevel of the needle shaft. A longer bevel can distribute stress, reduce resistance during the penetration process, and improve penetration efficiency (Montanari et al., 2023). Penetration efficiency led to clinical advantages, and *in vivo* and clinical studies have demonstrated low penetration force reduced the risk of mechanical retinal injury, the initiating inflammation, the requirement of intraoperative suturing and improved postoperative recovery after surgery (Nagpal et al., 2009; Kim et al., 2015; Wang et al., 2021; Wang et al., 2025; Lobo, 2012).

Design parameters such as tip geometry, outer diameter, tip length, and bevel length all feature prominently in determining trocar needles’ performance. A sharp tip is intended to reduce penetration resistance but an excessively small tip cross-sectional area can increase the risk of needle breakage in clinical practices (Choi, 2022), and requires higher cost as well as more rigorous demands on manufacturing precision. Besides, an overly long bevel may cause unnecessary tissue damage (Moreno et al., 2019). To address the issues, this study, using FEA, developed a multi-stage tapering needle with apex truncated and determined a reasonable bevel length to balance and optimize performance across various dimensions, such as higher cutting efficiency, minimal surgical damage, good needle tip strength, and intraoperative safety, thus establishing the optimal design parameter range for Sierra trocar needles.

Future studies can employ biomechanical experiments (such as micropuncture tests) and validation to physiological tissues to further validate the accuracy of the simulation models and optimize multiple parameters in order to develop new trocar needles. To elaborate further, more advanced materials or surface treatments could be used to enhance needle tip strength and wear resistance. Also, different tip geometries and bevel design could be further explored to better balance the relationship among penetration force, tissue damage, and cutting efficiency. Apart from this, given the unique characteristics and complexity of ocular anatomy, clinical studies could be taken to ensure the safety and effectiveness of new trocar needles in the further. Such studies will provide robust technical support for ophthalmic surgery developing toward greater precision and minimal invasiveness, which can reduce the risk of surgical complications and improve treatment outcomes as well as patients’ quality of life.

## Data Availability

The original contributions presented in the study are included in the article/supplementary material, further inquiries can be directed to the corresponding author.

## References

[B1] Choi H. S. (2022). Broken needle embedded in the body during vascular puncture. Healthcare 10 (8), 1436. 10.3390/healthcare10081436 36011093 PMC9408708

[B2] Davidović S. Babović S. Miljković A. Pavin S. Bolesnikov-Tošić A. Barišić S. (2024). Updates on treatment modalities for primary rhegmatogenous retinal detachment repair. Diagnostics 14 (14), 1493. 10.3390/diagnostics14141493 39061630 PMC11276041

[B3] DiMaio S. P. Salcudean S. E. (2003). Needle insertion modeling and simulation. IEEE Trans. Robotics Automation 19 (5), 864–875. 10.1109/tra.2003.817044

[B4] Foster W. J. Berg B. W. Luminais S. N. Hadayer A. Schaal S. (2022). Computational modeling of ophthalmic procedures. Am. J. Ophthalmol. 241, 87–107. 10.1016/j.ajo.2022.03.023 35358485 PMC9444883

[B5] Fujii G. Y. De Juan E. Humayun M. S. Pieramici D. J. Chang T. S. Awh C. (2002). A new 25-gauge instrument system for transconjunctival sutureless vitrectomy surgery. Ophthalmology 109 (10), 1807–1812. 10.1016/s0161-6420(02)01179-x 12359598

[B6] Gupta B. Neffendorf J. E. Williamson T. H. (2018). Trends and emerging patterns of practice in vitreoretinal surgery. Acta Ophthalmol. 96 (7), e889–e890. 10.1111/aos.13102 27213838

[B7] He Q. Huang J. He X. Yu W. Yap M. Han W. (2021). Effect of corneal incision features on anterior and posterior corneal astigmatism and higher‐order aberrations after cataract surgery. Acta Ophthalmol. 99 (7), e1027–e1040. 10.1111/aos.14778 33665973

[B8] Jiang L. Huang Y. Pan C. Ling J. Wen Y. (2016). “Research on insertion process of medical needle,” in *Proceedings of the 2016 3rd international conference on materials engineering, manufacturing technology and control* . 10.2991/icmemtc-16.2016.154

[B9] Kim M. J. Park K. H. Hwang J. M. Yu H. G. Yu Y. S. Chung H. (2007). The safety and efficacy of transconjunctival sutureless 23-gauge vitrectomy. Korean J. Ophthalmol. 21 (4), 201–207. 10.3341/kjo.2007.21.4.201 18063883 PMC2629884

[B10] Kim M. Park Y. S. Lee D. H. Koh H. J. Lee S. C. Kim S. S. (2015). Comparison of surgical outcome of 23-Gauge and 25-Gauge microincision vitrectomy surgery for management of idiopathic epiretinal membrane in pseudophakic eyes. Retina 35 (10), 2115–2120. 10.1097/IAE.0000000000000598 25978731

[B11] La Chapelle G. F. Swank H. A. Wessels M. E. Mol B. W. J. Rubinstein S. M. Jansen F. W. (2015). Trocar types in laparoscopy. Cochrane Database Syst. Rev. (12), CD009814. 10.1002/14651858.cd009814.pub2 26676093 PMC11227320

[B12] Lim L. T. El-Amir A. (2016). Minimally invasive sutureless day case vitrectomy surgery for retinal detachments, floaters, macular holes and epiretinal membranes – an experience from London, Windsor and reading. Adv. Eye Surg. 10.5772/62081

[B13] Lobo C. (2012). Pseudophakic cystoid macular edema. Ophthalmologica 227 (2), 61–67. 10.1159/000331277 21921587

[B14] Marques J. P. Azenha C. Figueira J. (2015). Microincision vitrectomy trocars – redefining surgical practices through a new range of applications. Eur. Ophthalmic Rev. 09 (01), 56. 10.17925/eor.2015.09.01.56

[B15] Mercanti A. Renna A. (2017). A review of microinvasive combined phaco-vitrectomy: recent technical advances. Ophthalmol. Ther. 6 (1), 49–54. 10.1007/s40123-017-0084-8 28357601 PMC5449303

[B16] Meyer C. H. Kaymak H. Liu Z. Saxena S. Rodrigues E. B. (2014). Geometry, penetration force, and cutting profile of different 23-gauge trocars systems for pars plana vitrectomy. Retina 34 (11), 2290–2299. 10.1097/iae.0000000000000221 25046392

[B17] Montanari M. Brighenti R. Terzano M. Spagnoli A. (2023). Puncturing of soft tissues: experimental and fracture mechanics-based study. Soft Matter 19 (20), 3629–3639. 10.1039/d3sm00011g 37161966

[B18] Moreno D. G. Pereira C. A. M. Sant Anna R. K. de Azevedo R. U. Savio L. F. Duarte R. J. (2019). Laparoscopic insertion of various shaped trocars in a porcine model. JSLS J. Soc. Laparoendosc. Surg. 23 (2), e2019.00002. 10.4293/jsls.2019.00002 31097906 PMC6476561

[B19] Nagpal M. Wartikar S. Nagpal K. (2009). Comparison of clinical outcomes and wound dynamics of sclerotomy ports of 20, 25, and 23 gauge vitrectomy. Retina 29 (2), 225–231. 10.1097/IAE.0b013e3181934908 19202426

[B20] Pang G. Wang C. Wang X. Li X. Meng Q. (2024). A review of human cornea finite element modeling: geometry modeling, constitutive modeling, and outlooks. Front. Bioeng. Biotechnol. 12, 1455027. 10.3389/fbioe.2024.1455027 39473927 PMC11518721

[B21] Sabti K. A. Raizada S. (2022). Novel surgical pathway for controlled access to the subretinal space: a case series. *Transl. Vis. Sci. and Technol.* 11 (4), 11. 10.1167/tvst.11.4.11 35416947 PMC9012894

[B22] Schutte S. van den Bedem S. P. W. van Keulen F. van der Helm F. C. T. Simonsz H. J. (2006). A finite-element analysis model of orbital biomechanics. Vis. Res. 46 (11), 1724–1731. 10.1016/j.visres.2005.11.022 16413594

[B23] Tanabe H. Kawasaki M. Ueda T. Yokota T. Zushi Y. Murayama R. (2020). A short bevel needle with a very thin tip improves vein puncture performance of peripheral intravenous catheters: an experimental study. J. Vasc. Access 21 (6), 969–976. 10.1177/1129729820920108 32372685

[B24] Wang Z. Bucher C. H. Van Linthout S. Tschöpe C. Schmidt-Bleek K. Duda G. N. (2021). Mechanobiological principles influence the immune response in regeneration: implications for bone healing. Front. Bioeng. Biotechnol. 9, 614508. 10.3389/fbioe.2021.614508 33644014 PMC7907627

[B25] Wang C. Liu M. Li Z. Zhang H. Wang Q. Zhu Y. (2025). Ophthalmic surgical robot for precise retinal puncture and drug delivery. Int. J. Extreme Manuf. 8 (1), 015503. 10.1088/2631-7990/ae00ff

[B26] Williamson T. H. (2021). in Vitreoretinal surgery. Editor Williamson T. H. (Cham: Springer International Publishing).

[B27] Zahedi S. A. Chizari M. Bader D. L. (2014). Evaluation of the friction coefficient, the radial stress, and the damage work during needle insertions into agarose gels. Proc. Institution Mech. Eng. Part H J. Eng. Med. 228 (3), 280–286. 10.1177/0954411913516350

